# Targeted muscle reinnervation surgery in a patient with neurofibromatosis type 1^✰^

**DOI:** 10.1016/j.jpra.2024.04.002

**Published:** 2024-04-07

**Authors:** Miss P. Keen, Y. Al-Ajam, N. Kang

**Affiliations:** a Imperial College School of Medicine, London, SW7 2AZ, UK; b Royal Free Hospital, London NW3 2QG, UK

**Keywords:** Targeted muscle reinnervation, Neurofibromatosis type 1, Phantom limb pain, Postamputation pain

Dear Sir/Madam,

A 58-year-old, right-hand-dominant woman with a history of neurofibromatosis Type 1 (NF-1), elected to undergo a left transradial amputation Figure 1. Over the preceding years, she had undergone multiple de-bulking procedures for a plexiform neurofibroma affecting the left hand and forearm. The lesion enlarged progressively and with each debulking procedure she lost more and more function and sensation in the hand. The hand became, insensate, painful, and non-functional, prompting the request for an amputation.

**Figure 1:**
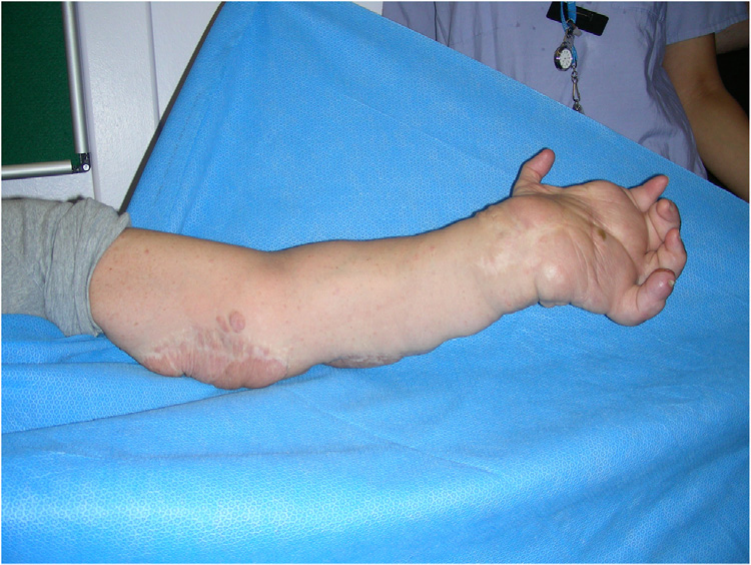
Left hand and forearm affected by a plexiform neurofibroma prior to de-bulking procedures, 2009.

At surgery in 2015, a transradial amputation was performed. The nerve stumps of the median and ulnar nerves were noted to be very enlarged. A standard traction neurectomy was performed for the nerves. Subsequent healing was uneventful.

Post-operatively, the patient reported strong symptoms of phantom limb pain (PLP). Therefore, a decision was made to perform a targeted muscle reinnervation (TMR) procedure. In 2019, the median and radial nerves were explored at the level of the antecubital fossa. The nerves were noted to be grossly enlarged Figure 2. When transected, there were few normal-looking axons in the proximal nerve stumps. Nevertheless, since there were suitable muscle targets for a TMR procedure (using the flexor digitorum superficialis as a target for median and the flexor digitorum profundus as a target for radial nerve), two transfers were performed.

**Figure 2:**
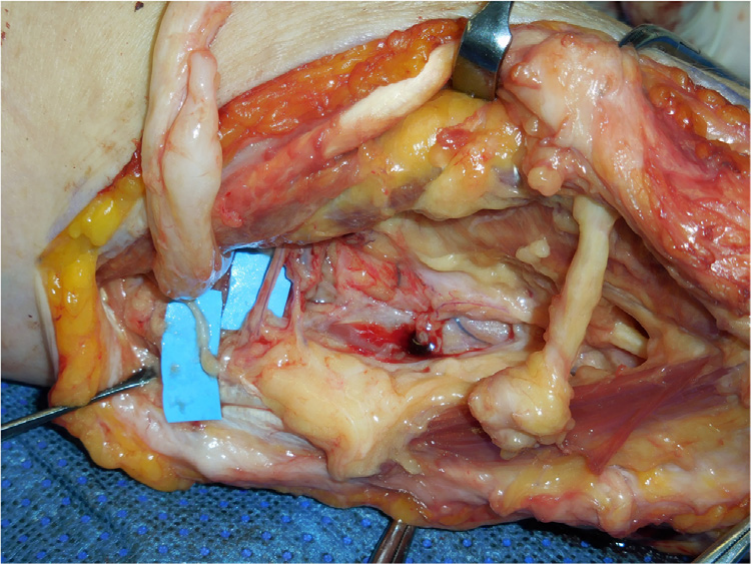
Exploring the limb: grossly engorged median and radial nerves at the level of the antecubital fossa.

At four years post-op, the patient reports that the TMR procedure has made no difference to her symptoms of PLP. This has raised questions about the value of TMR in a patient with NF-1.

Using the Patient Reported Outcomes Measurement Information System (PROMIS),^1^ the patient showed no reduction in pain interference for PLP. She also reported no change between pre- and postoperative numerical rating scale (NRS, 0–10) pain scores for baseline and pain episodes. She stated that she is dissatisfied with the outcomes of her TMR, and, in retrospect, would not choose to undergo the procedure again. She did not engage with any post-operative therapies including a psychologist, physiotherapist or occupational therapist and has also declined prosthetic fitting.

This is the first report of TMR surgery performed in a patient with NF-1. The outcome is not typical of those we have observed for non-NF-1 upper limb amputees undergoing TMR surgery. Amongst 14 upper limb amputees (mainly traumatic and one cancer) treated with TMR between 2012 and 2021, we observed 100% satisfaction with TMR and 100% of patients reported that they would undergo TMR surgery again if this were offered. Amongst this group, PROMIS pain interference scores reduced from a mean of 29 to 13 (*n* = [13]) and NRS baseline pain scores for PLP reduced from a mean of 5 to 2 (*n* = [14]).

This case report suggests that TMR surgery may not be of benefit for upper limb patients with NF-1. Although there is literature indicating that TMR reduces pain in NF-1 patients with a lower limb amputation,^2^ this evidence is lacking in the upper limb. We speculate that the procedure may work if the transfers are done at a level where the proximal nerve stumps are normal or if combined with a regenerative peripheral nerve interface (RPNI) procedure.^3^^,^^4^ Further research is warranted to validate our findings.

## Attributions

Dr Kang conceived of the idea for the article, performed the surgery, collated the photos, interpreted the data and helped to draft the article. Dr Al-Ajam helped to acquire and interpret the data, supervise writing of the article and reviewed the article. Miss Keen contributed to acquiring and interpreting the data, writing the article and put the information into a publishable format.

## Funding

None.

## Ethical approval

Informed consent was obtained from all patients involved.

## Declaration of competing interest

None.
